# Proteolytic Traits of Psychrotrophic Bacteria Potentially Causative of Sterilized Milk Instability: Genotypic, Phenotypic and Peptidomic Insight

**DOI:** 10.3390/foods10050934

**Published:** 2021-04-24

**Authors:** Stefano Morandi, Valentina Pica, Fabio Masotti, Stefano Cattaneo, Milena Brasca, Ivano De Noni, Tiziana Silvetti

**Affiliations:** 1 National Research Council, Institute of Sciences of Food Production, Via G. Celoria 2, 20133 Milan, Italy; stefano.morandi@ispa.cnr.it (S.M.); milena.brasca@ispa.cnr.it (M.B.); tiziana.silvetti@ispa.cnr.it (T.S.); 2 Department of Food, Environmental and Nutritional Sciences, Università degli Studi di Milano, Via G. Celoria 2, 20133 Milan, Italy; valentina.pica.94@gmail.com (V.P.); fabio.masotti@unimi.it (F.M.); stefano.cattaneo@unimi.it (S.C.)

**Keywords:** *Pseudomonas*, raw milk, AprX protease, casein, sterilized milk, liquid chromatography-mass spectrometry

## Abstract

The proteolytic traits of the psychrotrophic strains Pseudomonas poae LP5, Pseudomonas fluorescens LPF3, Chryseobacterium joostei LPR1, Pseudomonas fulva PS1, Citrobacter freundii PS37, Hafnia alvei PS46, and Serratia marcescens PS92 were initially investigated by phenotypic and genotypic approaches. Six strains elicited extracellular proteolytic activity, and five expressed the thermostable AprX or (likely) Ser1 enzymes. Then, the strains were inoculated (10^4^ CFU/mL) in microfiltered pasteurized milk and kept at 4 °C for five days. All of the strains reached 10^8^ CFU/mL at the end of storage and five produced thermostable extracellular proteolytic enzymes. The freshly inoculated samples and the corresponding samples at 10^8^ CFU/mL were batch-sterilized (131 °C, 30 s) and kept at 45 °C up to 100 days. The former samples did not gel until the end of incubation, whereas the latter, containing *P. poae*, *P. fluorescens*, *C. joostei*, *C. freundii*, and *S. marcescens,* gelled within a few days of incubation. The thermostable proteolytic activity of strains affected the peptidomic profile, and specific proteolyzed zones of β-CN were recognized in the gelled samples. Overall, the results confirm some proteolytic traits of psychrotrophic Pseudomonas spp. strains and provide additional insights on the proteolytic activity of psychrotrophic bacteria potentially responsible for sterilized milk destabilization.

## 1. Introduction

Sterilized milk may be defined as a homogenized product that has been heated to a temperature of 100 °C or above for a proper time so that it remains suitable for human consumption for a long period at room temperature. Thermal sterilization of milk requires time/temperature conditions capable of achieving a F_0_ value equal to or greater than three (EC Reg. 853/2004; EC Reg. 605/2010). Ultra-high temperature (UHT) treatment is a widespread continuous-flow process for milk sterilization and comprises direct or indirect heating at 135–150 °C for a short holding time (2–10 s). This treatment kills all microorganisms and bacterial spores, thus giving the final product a prolonged shelf life at ambient temperature [1,2]. The same technological effect is achievable through in-bottle sterilization, which, however, causes severe chemical changes and the loss of the nutritional value of sterilized milk.

The gelation of sterilized milk during storage (age gelation) is a major concern limiting its shelf-life, and much effort has been spent in understanding the causative factors and practices for preventing it. At least three different mechanisms have been suggested as responsible for age gelation: proteolysis by plasmin, proteolysis by bacterial proteases, and a physicochemical process via the aggregation of k-casein-depleted casein micelles. UHT treatment does not (completely) inactivate all native or exogenous proteases [3]. Indeed, UHT milk can undergo irreversible gelation upon the enzymatic degradation of bovine milk protein, especially casein (CN) [3,4]. For instance, the endogenous proteolytic “plasmin system” (plasmin/plasminogen and related activators/inhibitors) has been linked to the gelation of UHT milk [5]. This system has a certain thermal stability and its residual activity in UHT milk is a function of the severity of the thermal load [6]. For this reason, proteolysis by plasmin (EC 3.4.21.7) is reported to occur most often in direct UHT milk, and less often in the indirect-heated type, and to sequentially hydrolyze β-CN followed by α_S1_- and α_S2_-CN [7]. Another potential causative factor of gelation is the action of proteases produced by psychrotrophic bacteria during the refrigerated storage of raw milk to be heat-treated. Despite psychrotrophs being destroyed by (direct or indirect) UHT treatment, their extracellular proteases can continue their activities and cause UHT milk gelation after weeks/months of storage. Contrarily, most of these extracellular thermostable proteases are inactivated by in-bottle sterilization, which gives the final sterilized milk a longer shelf life [3]. Scientific and technological interest has been especially focused on extracellular alkaline metalloprotease AprX as a marker for psychrotrophs’ milk contamination and consequently, as a potential risk for UHT milk spoilage [8,9,10]. The *aprX* gene is present in several genera other than *Pseudomonas*, including *Citrobacter*, *Enterobacter,* and *Hafnia* [11]. Moreover, it has been reported that the AprX-induced gelation of UHT milk is mainly caused by the hydrolysis of κ-CN to produce “para-κ-casein-like” peptides [12]. This mechanism also has been pointed out for other exogenous caseinolytic enzymes [3]. Among psychrotrophic bacteria, *Pseudomonas* spp. are extensively studied because they are the most widespread. They are ubiquitous and can enter the milk via soil, vegetation, water, and equipment at farms and dairies [13]. Nevertheless, other bacteria commonly found in raw milk show the ability to produce heat-resistant extracellular metalloproteases. Some psychrotrophs (*Serratia*, *Aeromonas,* and *Enterobacter*) produce zinc-metalloproteases that can spoil UHT milk [14,15]. More recently, *Serratia* was frequently detected and characterized as a predominant raw-milk spoiler [14]. In this regard, *Serratia liquefaciens* FK01 produces two thermostable metallopeptidases, encoded by *ser1* and *ser2* genes, characterized by a variable and strain-dependent proteolytic activity [14,16].

Nowadays, there is great interest in gaining more knowledge on the proteolytic traits of psychrotrophic bacteria and on the potential impact for raw and UHT milk physical stability during (refrigerated) storage. This interest reflects the intensive research on these issues carried out so far [2,12,17]. In recent years, many authors studied the role played by *Pseudomonas* species and related (thermostable extracellular) enzymes on UHT milk instability [10,18,19]. Nonetheless, there is still room for further investigations into the proteolytic traits of other psychrotrophic species frequently recovered in bovine raw milk. Moreover, information about peptide patterns peculiar to the activity of psychrotrophic species on bovine milk proteins is still poor. Based on this, the present study was addressed to evaluate the occurrence of sedimentation/gelation during the storage of pasteurized milk inoculated with seven diverse psychrotrophic bacterial strains and the same samples after in-batch sterilization. To this purpose, the proteolytic traits of the psychrotrophic strains in milk samples were investigated by phenotypic (activity on milk agar plates and casein zymography), genotypic (presence/absence of *aprX* gene), and peptidomic (UPLC/HR-MS) approaches.

## 2. Materials and Methods

### 2.1. Bacterial Strains

The psychrotrophic strains Pseudomonas poae LP5, Pseudomonas fluorescens LPF3, Chryseobacterium joostei LPR1, Pseudomonas fulva PS1, Citrobacter freundii PS37, Hafnia alvei PS46, and Serratia marcescens PS92 were included in this study. These strains were previously isolated from raw milk samples [11] and belong to the strain collection of the “Institute of Sciences of Food Production of the National Research Council of Italy” (CNR-ISPA, Milan, Italy). Before the experiments, the strains were subcultured twice in brain heart infusion (BHI) broth (Biolife Italiana, Milan, Italy) for 24 h at 30 °C and were preserved in litmus milk (Biolife Italiana) at −18 °C.

### 2.2. Phenotypic and Genotypic Traits of Bacterial Strains

#### 2.2.1. Proteolytic Activity on Milk Agar Plate

Overnight cultures were streaked on milk agar plates (10% skim milk powder, 3% agar) and incubated at 30 °C for 48 h. The presence of a clear halo around the colonies was taken as indicative of extracellular proteolytic activity [11]. Tests were carried out in duplicate.

#### 2.2.2. Presence of the AprX Gene

Screening for the presence of the *aprX* gene in the test strains was carried out using the set of primers SM2F (5′-AAA-TCG-ATA-GCT-TCA-GCC-AT-3′)/SM3R (5′-TTG-AGG-TTG-ATC-TTC-TGG-TT-3′) with an amplification product of approximately 850 bp, as described by Marchand et al. [20]. DNA was extracted using the Microlysis kit (Aurogene, Rome, Italy) following the manufacturer’s instructions. The PCR reaction was performed in an Eppendorf Mastercycler nexus (Eppendorf AG, Hamburg, Germany), using the PCR Master Mix (2X) (Thermo Fisher Scientific, Walthman, MA, USA). The cycle parameters were 5 min at 95 °C for initial denaturation followed by 30 cycles with denaturation for 30 s at 95 °C, annealing for 30 s at 60 °C, extension for 1 min at 72 °C, and a final elongation step of 72 °C for 8 min.

#### 2.2.3. Expression of Extracellular Heat-Resistant Proteases

The cell-free supernatant (CFS) of the strains was prepared according to Marchand et al. and Stuknytė et al. [10,20]. Briefly, fresh cultures of psychrotrophic strains were inoculated in minimal salt medium (MSM) broth, with 2% of sterile skimmed milk, and incubated overnight at 30 °C. Subsequently, the strains were recovered in MSM with added CaCl_2_ (1 mM), then incubated at 21 °C for 6 h. Then, 10 mL of MSM were inoculated and incubated overnight at 21 °C. This procedure was repeated to favor the adaptation of the strains to the MSM. After 24 h of incubation at 21 °C in aerobic conditions, cells were harvested by centrifugation (10.000 g for 10 min at 4 °C). The supernatant was filter-sterilized (0.22 μm) and heat-treated at 95 °C for 8.45 min to select the heat-resistant proteases present in the CFS [20]. The heat-treated CFS (hereafter named HT-CFS) was used as a crude enzyme extract for casein zymography as described by Stuknytė et al. [10]. Tests were carried out in duplicate.

### 2.3. Proteolytic Activity of the Psychrotrophic Strains in Milk

#### 2.3.1. Preparation of Bacterial Inoculum

Bacterial inoculum preparation was performed following the procedure described by Stoeckel et al. [18]. Fresh cultures were centrifuged (3000 rpm, 15 min) and the cell pellets were washed twice with quarter-strength Ringer’s solution (Scharlau Microbiology, Barcelona, Spain). Pellets were then resuspended in microfiltered pasteurized milk (Parmalat, Collecchio, Italy), hereafter named pasteurized milk, and incubated at 2 °C for 3 days, allowing the bacteria to adapt to the milk medium and cold conditions. The cell counts after cold storage were about 10^8^ CFU/mL. Preparations were run in duplicate.

#### 2.3.2. Assessment of Thermostable Protease Production in Milk

An appropriate dilution of each adapted strain culture (see Section 2.3.1) was aseptically inoculated in pasteurized milk (Parmalat) to obtain a final concentration of 10^3^–10^4^ CFU/mL, and the inoculated samples were left at 4 °C for 5 days. Non-inoculated pasteurized milk, after refrigerated storage, was used as the control sample (CTRL pasteurized milk). At the end of cold storage, the production of thermostable proteases in milk by the tested strains was verified as described in Section 2.2.3. Tests were carried out in triplicate.

#### 2.3.3. Inoculation and Incubation of Experimental Milk Samples

**Figure 1:**
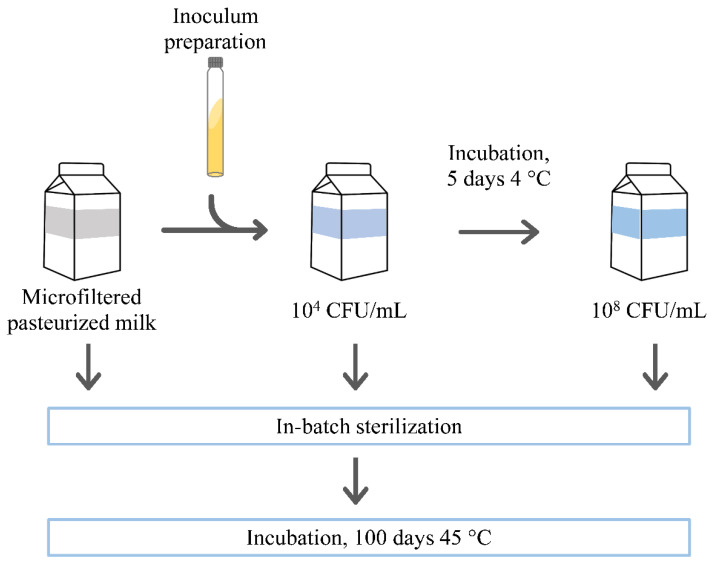
Preparation of experimental milk samples.

The inoculated experimental samples were prepared as depicted in Figure 1. An appropriate dilution of each adapted strain culture (see Section 2.3.1) was aseptically inoculated in pasteurized milk (Parmalat) to obtain a final concentration of 10^3^–10^4^ CFU/mL, and the inoculated samples were left at 4 °C for 5 days. Non-inoculated pasteurized milk, after refrigerated storage, was used as the control sample (CTRL pasteurized milk). Its total bacterial count was about 10^2^ CFU/mL, determined according to ISO 4833-1:2013 [21]. During refrigerated storage, inoculated samples were analyzed daily for bacterial content using the Petrifilm Aerobic Count Plate (3M, Pioltello, Italy) and visually controlled for the appearance of sedimentation or gelation.

Both the CTRL pasteurized milk and the inoculated samples at about 10^4^ CFU/mL were submitted to in-batch sterilization at 131 °C for 30 s using glass tubes immersed in a temperature-controlled oil bath (Labotech 2000, Cusago, Italy). After sterilization, tubes were promptly cooled in ice. The same treatment was applied to the inoculated samples at the end of 5 days of refrigerated storage when their bacterial count reached about 10^8^ CFU/mL. All of the heat-treated samples were checked for sterility by plating 100 µL of the sample on BHI agar (Biolife Italiana). Then, they were incubated at 45 °C for 100 days. During this period, the samples were constantly observed to assess whether milk sedimentation or gelation occurred. The non-inoculated sample, submitted to in-batch sterilization and 100 days of incubation at 45 °C, was used as the control sample (CTRL sterilized milk).

All of the tests were carried out in triplicate.

### 2.4. UPLC/HR-MS/MS Analysis, Peptide Identification, and Peptidomic Data Analysis

The proteolytic activity of strains was studied by assessing the peptidomic profiles of the inoculated pasteurized or in-batch sterilized milk samples through UPLC/HR-MS/MS. Before analysis, aliquots of inoculated pasteurized or in-batch sterilized samples and related non-inoculated CTRLs were ultrafiltered using an Omega modified polyethersulfone UF membrane (cut-off 10 kDa) in a Nanosep Advance device (Pall, Port Washington, NY, USA). The separation and identification of peptides was carried out by UPLC coupled to high-resolution tandem mass spectrometry (UPLC-MS/MS), using an Acquity UPLC module (Waters, Milford, MA, USA) interfaced with a Q Exactive hybrid quadrupole-Orbitrap MS through the HESI-II probe for electrospray ionization (Thermo Fisher Scientific, San Jose, CA, USA). Peptides were separated with an Acquity UPLC BEH130 C18 column (150 × 2.1 mm, 1.7 μm) (Waters, Milford, MA, USA) kept at 40 °C, and 0.1 mL 100 mL^−1^ formic acid (FA) in MilliQ-treated water (solvent A) and 0.1% FA in acetonitrile (solvent B) were used as eluents. A linear elution gradient was applied (5% to 50% of solvent B in 30 min) at a flow rate of 0.2 mL/min. The LC eluate was analyzed through HR-MS/MS, and a full scan and data-dependent tandem MS analysis (ddMS2) of the 5 most intense ions (Top5) was conducted. The ion source and interface conditions were set as previously reported [22]. Mass spectra were acquired over *m/z* range from 100–1500; ten of the most intense 1^+^–8^+^ charged ions detected in each spectrum underwent HCD fragmentation (data-dependent scan acquisition mode). The resolution was set at 70,000 and 17,500 for full scan and ddMS2 scan types, respectively. The AGC targets were 1 × 10^5^, and the maximum ion injection times were 110 ms. Peptide identification was done using the Proteome Discoverer 1.4 software (Thermo Fisher Scientific) and MS/MS spectra were searched using SequestHT against the database of *Bos taurus* (UniProt taxon ID 9913), considering their genetic variants. Automatic peak detection was performed with a signal-to-noise ratio setting of 4. A nonspecific enzyme cleavage pattern was defined, and dynamic modifications were the phosphorylation of serine and threonine; deamidation of asparagine, glutamine and arginine; oxidation of methionine; and cyclisation of an N-terminal glutamine to pyro-glutamic acid. Mass error tolerance for precursor ions was 5 ppm and for fragment ions was 0.02Da. A strict false discovery rate of peptide identification was set (FDR = 0.01).

## 3. Results and Discussion

### 3.1. Phenotypic and Genotypic Characterization of Psychrotrophic Strains

The proteolytic activity of the single psychrotrophic strains was investigated through the phenotypic and genotypic approaches. Firstly, the ability to produce proteolytic extracellular enzymes was assessed on milk agar plates and, apart from *H. alvei,* all of the strains produced lytic halos (Table 1). In agreement with our results, *H. alvei* strains previously investigated by Ercolini et al. did not display proteolytic activity on milk agar, both at 7 and 20 °C [23].

Further investigation was carried out by submitting the HT-CFS of the strains to casein zymography with the aim of assessing the caseinolytic activity related to extracellular thermostable enzymes [10]. Indeed, the heat treatment applied to CFS promotes the depletion of any pre-existing heat-labile endogenous or exogenous proteases and, therefore, favors the selection of thermostable proteases. Casein zymography showed that the HT-CFS of *P. poae, P. fluorescens*, *C.* *joostei*, *C. freundii*, and *S. marcescens* contain a thermostable protease with an estimated molecular weight of about 45 kDa (data not shown). These results agreed with those of Baglinière et al. and Zarei et al., who reported that *Pseudomonas* and *Serratia* strains isolated from bovine raw milk produce thermostable caseinolytic enzymes with an approximate molecular weight of 45–48 kDa [24,25].

PCR analysis showed the presence of the *aprX* gene in *P. poae, P. fluorescens, C. joostei,* and *C. freundii* (Table 1). This gene was not detected in *P. fulva, S. marcescens,* and *H. alvei,* but only the latter strain did not show proteolytic activity on milk agar plates. The zymogram of the HT-CFS from *P. fulva* did not reveal the presence of heat-resistant proteases (data not shown), thus suggesting that its proteolytic activity on agar plate involved heat-labile extracellular enzymes. A similar finding was recently reported by Zarei et al., who showed the presence of non-heat-resistant proteases in different strains belonging to the *P. fluorescens* group [25]. The caseinolytic activity of the HT-CFS from *S. marcescens* was likely related to the action of the thermostable protease Ser1, as suggested by the presence of a proteolytic band of approximately 45–48 kDa in the zymogram (data not shown) [16]. Nonetheless, the production of Ser1 by *S. marcescens* has been not reported so far. This enzyme belongs to the serralysin family, and it was detected for the first time in *S. liquefaciens* isolated from bovine raw milk [24]. As for AprX, the primary structure of Ser1 shows the presence of the consensus motives HEXXHXUGUXH and GGXGXDXUX for the binding of Zn^2+^ and Ca^2+^, respectively. Moreover, Ser1 proteases contain the ABC exporter motif (DXXX), indicating they are extracellular enzymes [26,27].

Overall, as summarized in Table 1, the results showed 6 out of 7 studied strains to elicit extracellular proteolytic activity, but only 5 of them expressed the AprX or, likely for *S. marcescens*, the Ser1 enzyme. These findings stress the importance of differentiating proteolytic psychrotrophic strains capable of producing thermostable proteases and hence, to potentially affect sterilized milk stability during storage [8,20,28,29].

### 3.2. Characterization of Proteolytic Activity of the Psychrotrophic Strains in Pasteurized Milk Prior to and after In-Batch Sterilization

To assess the proteolytic activity of the test strains in milk, aliquots of pasteurized milk were inoculated with the single psychrotrophic strains to obtain a final concentration of about 10^4^ CFU/mL and then were incubated at 4 °C for five days (Figure 1). Microfiltered pasteurized milk was chosen as an almost bacteria-free growth medium, thus minimizing the role of endogenous bacteria (and related intracellular enzymes) to proteolytic phenomena occurring in the experimental milk samples. This sample was used as the control and, at the end of refrigerated incubation, its total bacterial count did not change. Contrarily, in the inoculated pasteurized milk samples, all of the considered strains reached the stationary growth phase within 3–4 days, growing about one log per day. *P. fluorescens* and *H. alvei* exhibited the fastest growth rate, achieving about 10^8^ CFU/mL after 2–3 days, while the other strains reached this concentration at the end of refrigerated storage. Similar growth behaviors were observed by Stoeckel et al. and Zhang et al. for different strains belonging to the *Pseudomonas* species [18,19]. Despite this, none of the strains affected the physical stability of pasteurized milk until the end of the refrigerated storage (Table 2). At this time point, the CTRL pasteurized milk and the inoculated samples were heated at 95 °C for 8.45 min and submitted to casein zymography to evaluate the production of thermostable proteases during refrigerated storage of milk [20]. The CTRL pasteurized milk sample did not show thermostable activity. Thermostable AprX or Ser1 proteases production in milk was confirmed for five out of seven strains (data not shown). Weaker lysis of CN was observed for *P. poae* and *C. freundii*, while *P. fluorescens, C. joostei,* and *S. marcescens* evidenced stronger activity. As expected, the samples inoculated with *P. fulva* and *H. alvei* did not show any thermostable activity. In this regard, it has been reported that production of extracellular protease by *Pseudomonas* strains is a common feature during the log phase, and it is highest when they reach the stationary phase of bacterial growth [30,31]. Moreover, the temperature-dependent production of heat-stable proteases was demonstrated for *Pseudomonas* spp. [32]; recently, Wang et al. reported that the AprX-producing *P. fluorescens* W3 had a lower ability to secrete extracellular proteases at 4 °C than at 30 °C [33]. The same authors observed that *P. fluorescens* W3 grew slower and showed lower proteolytic activity under low temperature conditions. This finding, and the short incubation period, could explain why, at the end of refrigerated storage, none of the inoculated pasteurized samples had undergone gelation.

The proteolytic activity of the psychrotrophic strains was also studied after in-batch sterilization of pasteurized inoculated milk (Figure 1). To this purpose, the pasteurized samples were batch-sterilized just after inoculation at 10^4^ CFU/mL and later when their bacterial count reached 10^8^ CFU/mL at the end of refrigerated storage. The lowest bacterial count represents a conceivable level of psychrotrophic contamination in milk, whereas the highest one was adopted to stress the proteolytic phenomena potentially associated with milk contamination by psychrotrophic bacteria. The applied in-batch treatment (131 °C for 30 s) corresponded to a F_0_ value of 4.9. This value fulfills the minimal heat load for sterilizing drinking milk (EC Reg. 853/2004; EC Reg. 605/2010) and is in the range (5–6) of F_0_ values recommended for UHT treatment of good quality milk [34]. This treatment inactivated the residual plasmin activity in the sterilized samples but kept active the thermostable proteases. Indeed, according to Zhang et al., after treatment at 130 °C for 2 min, more than 41.7% of the protease activity of *P. fluorescens* BJ-10, originally isolated from raw milk, was maintained [35]. In-batch sterilized samples were incubated at 45 °C to favor proteolytic phenomena and, over 100 days of incubation, physical changes of samples were assessed by eye and classified as reported by Anema [3]. Sedimentation was judged as a dense compact layer of protein material only present at the bottom of the tube. Gelation was stated when a three-dimensional protein network formed, causing the loss of milk fluidity and occupying the entire volume of the tube.

The samples sterilized at initial 10^4^ CFU/mL bacterial count did not gel until the end (100 days) of incubation. Only a slight sediment formed in samples inoculated with *C. joostei* and *C. freundii* after 4–5 days of incubation (Table 2). Stoeckel et al. and Zhang et al. highlighted that sedimentation occurred before the gelling phenomenon in UHT milk samples containing *Pseudomonas* strains [18,19]. To the best of our knowledge, there are no data on sediment/gel formation by *Citrobacter* and *Hafnia* in literature, and little evidence is available for *Chryseobacterium* and *Serratia* [24,36]. In-batch sterilization was also carried out on inoculated samples at the end of refrigerated storage. As expected, samples inoculated with *P. fulva* and *H. alvei* did not evidence any physical change. Contrarily, samples containing *P. poae, P. fluorescens*, *C. joostei*, *C. freundii*, and *S. marcescens* showed gelation within two days of incubation at 45 °C (Table 2).

These gels appeared to be a tight protein network peculiar to the action of bacterial proteases. These differences may be mostly attributed to the different proteolytic potential resulting from diverse levels of AprX expression during the refrigerated storage of milk [8]. Indeed, the *aprX* gene results are highly conserved within bacterial species but are, however, heterogeneous among the psychrotrophic species [14]. Similar results were reported by D’Incecco et al. who observed gel formation in milk samples containing dead cells (~10^8^ CFU/mL) of different *Pseudomonas* strains after 1–4 days of incubation at 40 °C [36]. Different studies highlighted that UHT milk gelation is strictly related to psychrotrophic bacteria content in starting milk, since maximum thermostable protease expression is achieved when the psychrotrophic strains reach the late exponential phase of growth (10^7^–10^8^ CFU/mL) [3,19,37]. In this regard, Alves et al. and Wang et al. reported that *P. fluorescens* W3 and *P. fluorescens* 07A expressed higher level of (thermostable) proteases at refrigerated conditions to compensate for the decreased enzymes’ activity [33,38]. This could explain why the growth of psychrotrophic bacteria in milk kept at low temperature for a long time can be detrimental for the physical stability of the derived sterilized milk, but not for the starting refrigerated raw milk.

Overall, the present results showed that the tested strains grow at different rates in refrigerated pasteurized milk, and some of them produce thermostable extracellular proteolytic enzymes during storage. In addition, the thermostable proteolytic activity of psychrotrophic bacteria differently affects the physical stability of in-batch sterilized milk. The formation of sediment was observed in two of the samples with low bacterial content (10^4^ CFU/mL), while gelation occurred in all samples inoculated with strains capable of producing thermostable proteases as a result of their development up to 10^8^ CFU/mL.

### 3.3. Peptidomic of Milk Samples Inoculated with the Studied Psychrotrophic Strains

**Figure 2:**
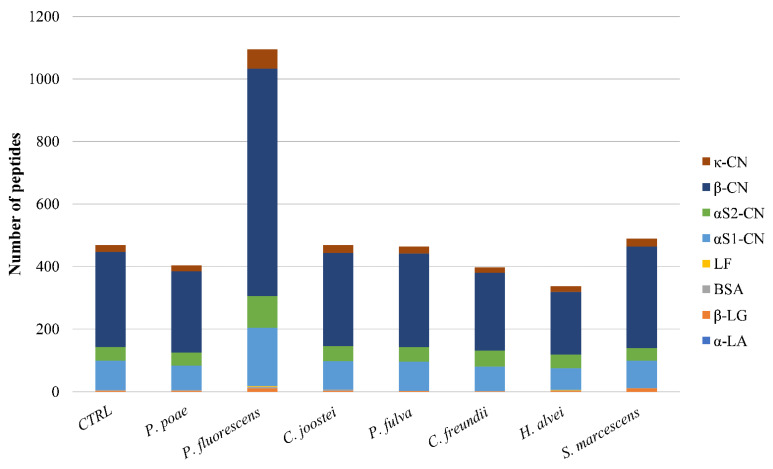
Number and precursor proteins of the peptides identified in CTRL pasteurized milk (CTRL), and in pasteurized milk samples inoculated with the psychrotrophic strains, after storage at 4 °C for five days. κ-CN, κ-casein; β-CN, β-casein; α_S2_-CN, α_S2_-casein; α_S1_-CN, α_S1_-casein; LF, lactotransferrin; BSA, bovine serum albumin; β-LG, β-lactoglobulin; α-LA, α-lactalbumin.

The qualitative peptidomic analysis was initially addressed to study the proteolytic activity of the studied strains inoculated in pasteurized milk and maintained in refrigerated conditions. At the beginning of incubation, the non-inoculated pasteurized milk contained 277 unique peptides, and most of them were from α_S_-CN and β-CN (data not shown). The number of peptides increased to 469 after five days of storage at 4 °C, mainly due to the augment of peptides released from β-CN (Figure 2). This phenomenon was likely sustained by plasminolysis, which mainly induces the hydrolysis of β-CN [7]. Except for the sample containing *P. fluorescens*, the number of peptides in inoculated samples and the precursor proteins they released therefrom almost overlapped that of CRTL pasteurized milk (Figure 2). At the end of storage, the sample inoculated with *P. fluorescens* contained about 1100 peptides and, importantly, it presented the highest proportion (16%) of peptides with MW > 3 kDa (data not shown). This type of peptide ranged between 2–8% of total identified species in the other inoculated samples. Overall, at the end of refrigerated storage, the seven inoculated samples and the CTRL pasteurized milk shared about 200 unique peptides, most of them (60%) having been released from β-CN.

**Figure 3:**
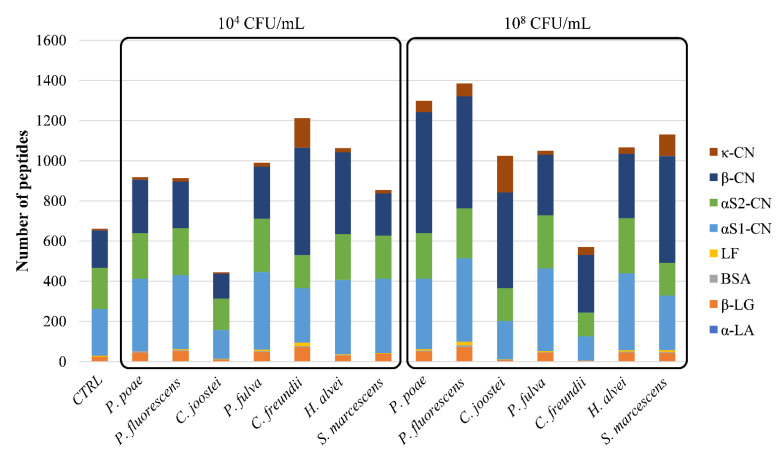
Number and precursor proteins of the peptides identified after incubation at 45 °C for 100 days in CTRL sterilized milk (CTRL) and in milk samples sterilized when the psychrotrophic count of pasteurized milk reached 10^4^ or 10^8^ CFU/mL. κ-CN, κ-casein; β-CN, β-casein; α_S2_-CN, α_S2_-casein; α_S1_-CN, α_S1_-casein; LF, lactotransferrin; BSA, bovine serum albumin; β-LG, β-lactoglobulin; α-LA, α-lactalbumin.

Peptidomic analysis on in-batch sterilized samples was carried out when milk gelation/sedimentation occurred or at the end of incubation (100 days at 45 °C) if physical changes did not arise. The CTRL sterilized sample did not gel or sediment during incubation and, at the end, it contained about 661 peptides mostly released from α_S1_-CN > α_S2_-CN > β-CN (Figure 3). Peptides smaller than 3 kDa were the most abundant (87%). Gelation did not occur in samples in-batch sterilized when the bacterial count of milk was 10^4^ CFU/mL (Table 2). After 4–5 days, slight sedimentation occurred in sterilized samples inoculated with *C. joostei* and *C. freundii* (Table 2), which contained about 450 and 1200 peptides, respectively (Figure 3). The latter sample presented a larger proportion of species with MW < 1 kDa (49%) with respect to the sample inoculated with *C. joostei* (8%). Contrarily, sterilized milk samples with *P. poae*, *P. fluorescens, P. fulva, H. alvei,* and *S. marcescens* at 10^4^ UFC/mL did not show visible destabilization within 100 days of incubation at 45 °C and shared similar peptidomic profiles (Figure 3). In these samples, about 1000 peptides were revealed, and those in the range 1–3 kDa (60–69%) prevailed (data not shown). Based on these findings, the number of released peptides did not demonstrate a reliable index for predicting or describing the physical destabilization of sterilized milk.

After 1 or 2 days of incubation, gelation was observed in samples that were heat-treated when the bacterial count of *P. poae*, *P. fluorescens*, *C. joostei*, *C. freundii,* and *S. marcescens* was 10^8^ CFU/mL (Table 2). All of these strains were phenotypically and genetically capable of eliciting proteolytic activity through extracellular thermostable enzymes based on *aprX* gene detection and casein zymography (Table 1). Moreover, the same strains expressed the AprX or, likely for *S. marcescens,* Ser1 enzymes, when inoculated in pasteurized milk kept under refrigerated conditions (data not shown). The gelled samples contained a different number (about 600–1400) of peptides coming from β-CN > α_S_-CN > κ-CN (Figure 3). These findings agree with those of Baglinière et al. who demonstrated that the AprX protease from *P. fluorescens* hydrolyzed all CNs with a preference for β-CN in UHT milk [39,40]. Contrarily, other studies showed the extracellular thermostable proteases of *Pseudomonas* spp. strains to preferentially hydrolyze κ-CN along with β-CN [28,29,41]. In particular, the thermostable protease AprX was demonstrated to elicit a chymosin-like activity through cleaving the peptidic bonds at Phe_105_–Met_106_, Ser_104_–Phe_105_, Met_106_–Ala_107_, and Ala_107_–Ile_108_ in κ-CN and releasing caseinomacropeptide (CMP, κ-CN (106-169)) or pseudo-CMP (κ-CN (105-169), (107-169), (108-169)) [36,42]. None of these (pseudo-)CMPs were found in the sedimented samples, which were sterilized when the bacterial count of *C. joostei* and *C. freundii* was 10^4^ CFU/mL. Despite this, 12% of the total peptides released from κ-CN when *C. freundii* was used for the inoculum. The pseudo-CMP κ-CN f(108-169) was detected only in the gelled sterilized sample with *P. poae* (10^8^ CFU/mL). All of the other samples (not sedimented or gelled) did not contain the mentioned (pseudo-)CMPs. Likely, chymosin-like activity is not the sole proteolytic phenomenon potentially responsible for sterilized milk gelation, and the specificity of enzymatic cleavage in other sites of CN sequences is paramount for physical changes occurring in sterilized milk, at least in the studied samples.

Pasteurized milk samples with *P. fulva* or *H. alvei,* which were in-batch sterilized at a bacterial concentration of 10^4^ or 10^8^ CFU/mL, did not show milk sedimentation or gelation within 100 days of incubation. These strains did not possess the *aprX* gene and the peptidomic profiles of the related sterilized samples were similar (Figure 3). These findings suggest that AprX and Ser1 proteases likely acted as the major proteolyzing enzymes in the studied samples and played a paramount role in sterilized milk gelling.

Overall, irrespective of the strains and the inoculum rate studied, β-CN was the most proteolyzed protein (36% of total identified peptides), followed by α_S1_-CN (32%), α_S2_-CN (22%), κ-CN (5%), and β-lactoglobulin (4%). The peptides released from α-lactalbumin, bovine serum albumin, and lactotransferrin were represented by less than 1%. Peptides in the range 1–3 kDa were the most abundant in each sample (54 to 73%), generally followed by peptides smaller than 1 kDa (6 to 49%), then by peptides >3 kDa (2 to 25%). This result was not confirmed in the case of sterilized milk containing 10^4^ CFU/mL of *C. joostei* (LPR1) and 10^8^ CFU/mL of *C. freundii* before heat treatment, where the peptides <1 kDa were very limited in number (8% and 6%, respectively). These two samples were also characterized by a lower number of peptides (about 450 and 600, respectively), compared to other sterilized samples, suggesting scarce proteolysis.

**Figure 4:**
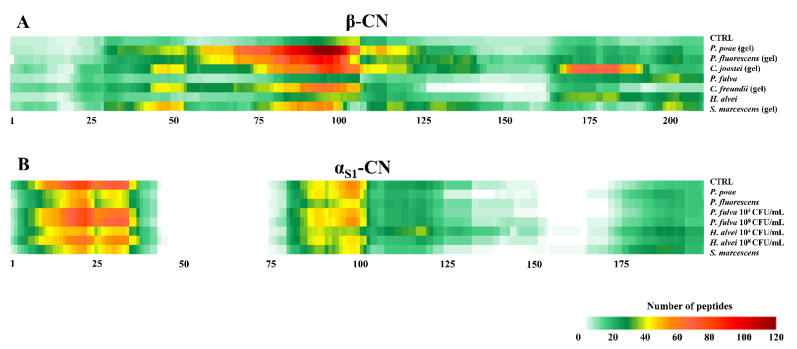
Qualitative visualization of proteolytic patterns of: (**A**), β-CN in sterilized samples. From the top: CTRL sterilized milk, sterilized milk inoculated (10^8^ CFU/mL) with *P. poae, P. fluorescens, C. joostei, P. fulva, C. freundii, H. alvei,* and *S. marcescens*; (**B**) α_S1_-CN in sterilized samples, which did not show sedimentation or gelation during incubation at 45 °C for 100 days. From the top: CTRL sterilized milk, sterilized milk with *P. poae, P. fluorescens, P. fulva, H. alvei,* and *S. marcescens.* (CFU/mL: the bacterial count of pasteurized milk before in-batch sterilization). The color code from green to red indicates the frequency at which an amino acid was identified within the β-CN and α_S1_-CN sequences and gaps are regions where no peptides were identified.

Despite different numbers and dimension distributions of peptides, a specific proteolytic pattern can be recognized along the β-CN sequence in gelled samples (Figure 4). Protein region 75–100 released most of peptide species in all of the gelled samples and likely this degradation could be involved in milk gelation. Other regions were less proteolyzed, like the N-terminus, including the phosphorylated cluster β-CN (17–21). Also Bagliniere et al. reported that, in UHT milk contaminated with different *P. fluorescens* strains, β-CN was strongly hydrolyzed throughout its sequence except in the N-terminus (1–25) [39]. In all of the samples that did not gel or sediment, the peptide pattern resembled that of CTRL sterilized milk. Peptides from α_S1_-CN prevailed and the N-terminus region was mostly hydrolyzed (Figure 4). No peptides were identified in correspondence with the phosphorylated cluster α_S1_-CN (66–70).

## 4. Conclusions

The outcomes of the present study confirm some proteolytic traits of psychrotrophic *Pseudomonas* spp. strains potentially causative of sterilized milk instability. They also add new knowledge on the proteolytic activity of other psychrotrophic bacteria responsible for UHT milk destabilization as well. Nonetheless, the results show that the susceptibility of sterilized milk to gel is not related solely to the extent of proteolysis as evidenced by the number of released peptides. In this regard, the study suggests that the level of psychrotrophs’ contamination is a crucial but not sufficient factor to cause gelation in sterilized milk. Indeed, the peptidomic profiles here revealed in milk samples seem somehow similar among the inoculated strains, yet differently effective in promoting CN destabilization and the gelation/sedimentation of sterilized milk. Nonetheless, the thermostable proteolytic activity exerted on β-CN seemed peculiar to gelled sterilized samples. Despite this, the (bio)chemical and physical phenomena involved in the age gelation of sterilized milk are far from being completely understood and still need proof of evidence.

## Figures and Tables

**Table 1:** Genotypic and phenotypic traits of the psychrotrophic strains.

Genus	*Pseudomonas*	*Pseudomonas*	*Chryseobacterium*	*Pseudomonas*	*Citrobacter*	*Hafnia*	*Serratia*
Species	*poae*	*fluorescens*	*joostei*	*fulva*	*freundii*	*alvei*	*marcescens*
Strain	LP5	LPF3	LPR1	PS1	PS37	PS46	PS92
Proteolytic activity on milk agar ^1^	+	+	+	+	+	-	+
*aprX* gene ^2^	+	+	+	-	+	-	-
Extracellular thermostable proteases	AprX	AprX	AprX	-	AprX	-	+ (Ser1)

^1^ + active, - not active. ^2^ + present, - absent.

**Table 2:** Physical phenomena occurring in pasteurized samples inoculated with the psychrotrophic strains and in the in-batch sterilized samples.

	Genus	*Pseudomonas*	*Pseudomonas*	*Chryseobacterium*	*Pseudomonas*	*Citrobacter*	*Hafnia*	*Serratia*
	Species	*poae*	*fluorescens*	*joostei*	*fulva*	*freundii*	*alvei*	*marcescens*
	Strain	LP5	LPF3	LPR1	PS1	PS37	PS46	PS92
Pasteurized milk ^1^	Sedimentation ^3^	no	no	no	no	no	no	no
	Gelation ^3^	no	no	no	no	no	no	no
In-batch sterilized milk ^2^	Sedimentation ^3^	no	no	yes (10^4^ CFU/mL)	no	yes (10^4^ CFU/mL)	no	no
	Gelation ^3^	yes ^4^ (10^8^ CFU/mL)	yes ^4^ (10^8^ CFU/mL)	yes ^4^ (10^8^ CFU/mL)	no	yes ^4^ (10^8^ CFU/mL)	no	yes ^4^ (10^8^ CFU/mL)

^1^ Pasteurized milk inoculated at 10^3^–10^4^ CFU/mL and incubated for five days at 4 °C. ^2^ Milk samples were in-batch sterilized when the bacterial count reached 10^4^ CFU/mL or at the end of refrigerated storage when the bacterial count increased up to 10^8^ CFU/mL. After in-batch sterilization, samples and related CTRL were incubated for 100 days at 45 °C. ^3^ Checked by visual assessment (in brackets is the bacterial count of the pasteurized milk sample before in-batch sterilization). ^4^ Within 2 days.

## Data Availability

Data reported in this manuscript will be available upon request.
